# Comparison of bacterial communities in soil samples with and without tomato bacterial wilt caused by *Ralstonia solanacearum* species complex

**DOI:** 10.1186/s12866-020-01774-y

**Published:** 2020-04-14

**Authors:** Ying Zhang, Anna Hu, Jianuan Zhou, Wenfei Zhang, Peng Li

**Affiliations:** 1 Ministry of Education Key Laboratory for Ecology of Tropical Islands, College of Life Sciences, Hainan Normal University, Haikou, 571158 China; 2 Guangdong Laboratory for Lingnan Modern Agriculture, Integrative Microbiology Research Centre, Guangdong Province Key Laboratory of Microbial Signals and Disease Control, College of Agriculture, South China Agricultural University, Guangzhou, 510642 China

**Keywords:** Biological control, Conducive soils, Interaction network, Rhizosphere bacteria, *Ralstonia solanacearum*, Suppressive soils, Tomato

## Abstract

**Background:**

*Ralstonia solanacearum* is one of the most notorious soil-borne phytopathogens. It causes a severe wilt disease with deadly effects on many economically important crops. The microbita of disease-suppressive soils are thought that they can contribute to the disease resistance of crop plants, thus, evaluation of the microbial community and their interaction characteristics between suppressive soil (SS) and conducive soil (CS) will help to understand resistance mechanism. To do this, the bacterial community structure, correlation analysis with soil chemical properties, interaction network of SS (nearly no disease in three years), and CS (suffered heavy bacterial wilt disease) were analyzed.

**Results:**

A higher bacterial community diversity index was found in SS, the relative abundance of *Nocardioides, Gaiella* and *norank_f_Anaerolineaceae* were significantly more than that of the CS. Moreover, the relative abundance of main genera *Bacillus*, *norank_o*_*Gaiellales*, *Roseiflexus*, and *norank_o_Gemmatimonadaceae* were significantly more than that of the CS. Redundancy analysis at the genus level indicated that the available phosphate played a key role in the bacterial community distribution, and its role was negatively correlated with soil pH, organic matter content, alkali-hydrolyzable nitrogen, and available potassium contents. Interaction network analysis further demonstrated that greater diversity at the genus level existed in the SS network and formed a stable network. Additionally, the species of *Mycobacterium*, *Cyanobacteria*, and *Rhodobiaceae* are the key components that sustain the network stability. Seven clusters of orthologous groups exhibited significant differences between SS and CS. Moreover, 55 bacterial strains with distinct antagonistic activities to *R. solancearum* were isolated and identified from the healthy tomato plant rhizosphere soil of the CS.

**Conclusions:**

Our findings indicate that the bacterial diversity and interaction network differed between the CS and SS samples, providing a good foundation in the study of bacterial wilt.

## Background

Tomato (*Solanum lycopersicum*) is one of the most commonly cultivated vegetables in the world; however, the soil-borne disease caused by *Ralstonia solanacearum* species complex (RSSC) is a serious threat to tomato production. Moreover, more than 200 hosts in 54 botanical families can be infected by the members of RSSC [1], leading to severe economic and social impact worldwide [2, 3]. RSSC can exist in the soil for many years, meaning that infected fields are less likely to successfully cultivate susceptible plants [4].

Disease-suppressive soils are exceptional ecosystems. It is widely thought that the soil microbiota contribute to the disease resistance of crop plants [5, 6]. In recent years, increasing evidences have been reported for suppressing several soil-borne pathogens causing *Fusarium* wilt [7], potato common scab [8], damping-off disease [9], sugar beet wilt [10], and bacterial wilt [11]. In addition, further research showed that the diversity of the soil microbial community is particularly crucial for maintaining the disease suppressing capacity, which can affect the colonization success of additional species [12, 13]. Some beneficial bacteria have been introduced into soils to increase the microbial community diversity or enhance the resistance to RSSC [14, 15].

The tomato cultivar “Lingshui cherry tomato” which is well known for its unique flavor, is cultivated in large area and it is honored as China’s national geographical indication products. However, due to long-term continuous monoculture, large areas of tomato fields suffered severe bacterial diseases in recent decades. Despite consistent tomato culturing and management patterns, the tomato grew well in some fields. In this study, we aimed to evaluate the differences in soil bacterial diversity and soil chemical properties between suppressive soil (SS, nearly no disease in 3 years) and the conducive soil (CS, suffered heavy bacterial wilt disease), with the goal of suppressing plant disease and protecting plant health.

Herein, a high-throughput sequencing technology was used to explore the differences in bacterial diversity between SS and CS. In addition, the soil chemical properties, bacterial community composition, network analysis and clusters of orthologous groups (COGs) were analyzed. Importantly, 55 bacterial strains with excellent antagonistic ability against *R. solanacearum* from the rhizosphere soil of healthy tomato plants were isolated and identified. This information provides more biocontrol resources for the control of bacterial wilt.

## Results

### Bacterial diversity assessment of the SS and CS samples

**Fig. 1:**
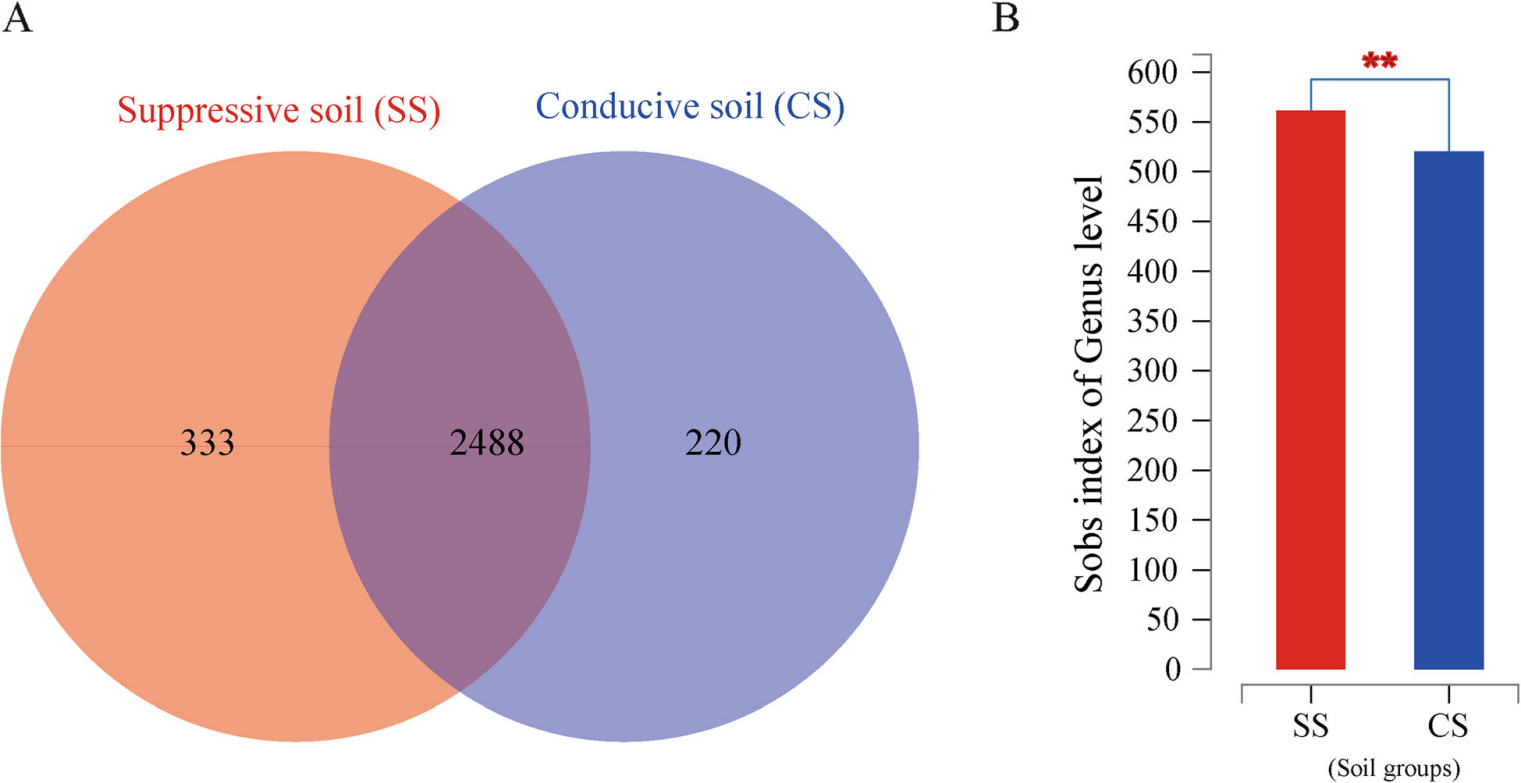
Venn analysis of shared and unique OTUs between suppressive soil (SS) and conducive soil (CS) samples **a**. OTUs defined by 97% sequence similarity and Student’s *t*-test of OTU level between suppressive soil (SS) and conducive soil (CS) **b**. **p ≤ 0.01

In order to determine whether the microbial community functioned to sustain tomato health, we identified the difference in disease incidence of tomato plants cultivated under four growing conditions: SS, CS, CS with heat treatment, and sterile nutrient soil inoculated with the *R. solanacearum* suspension. The results demonstrated that all of the tomato plants showed wilt symptoms after 4 days of *R. solanacearum* suspension inoculation. The tomato plants cultivated in CS showed typical wilt symptoms and nearly 80% of plants wilted. Tomato plants grown in SS and the heat-treated soil exhibited no plant wilt (supplemental information, S1). The bacterial diversity of soil samples collected from SS and CS was assessed using phylotype taxonomy. The results revealed a total of 3041 operational taxonomic units (OTUs). The core OTU number was 2488, and the SS and CS exhibited 330 and 220 unique OTUs, respectively (Fig. 1a). Furthermore, the result of Student’s *t*-test indicated that the Sobs index of the OTU level of SS and CS samples was significant (*p* = 0.002216; Fig. 1b).

### Main bacterial composition of SS and CS

**Fig. 2:**
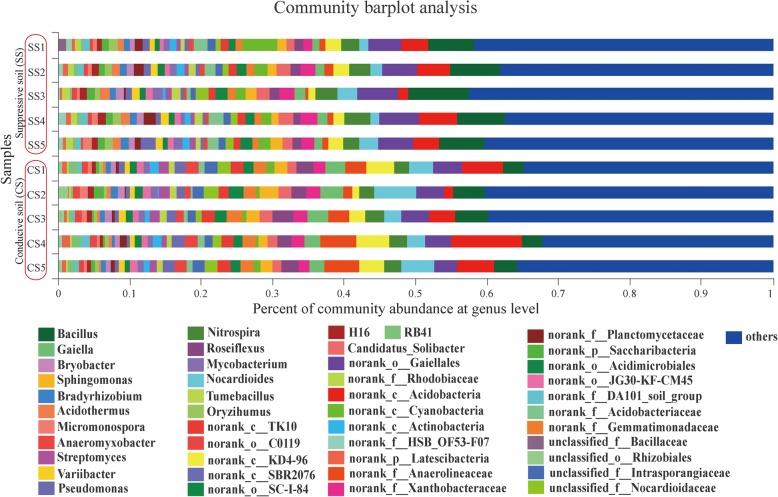
Community bar plot analysis of five suppressive soil (SS) samples (SS1, SS2, SS3, SS4, and SS5) and five conducive soil (CS) samples (CS1, CS2, CS3, CS4, and CS5) at the genus level. “Others” indicates genera with the relative abundance lower than 0.1%. The *x*-axis of the column is the percentage relative abundance at the genus level

**Fig. 3:**
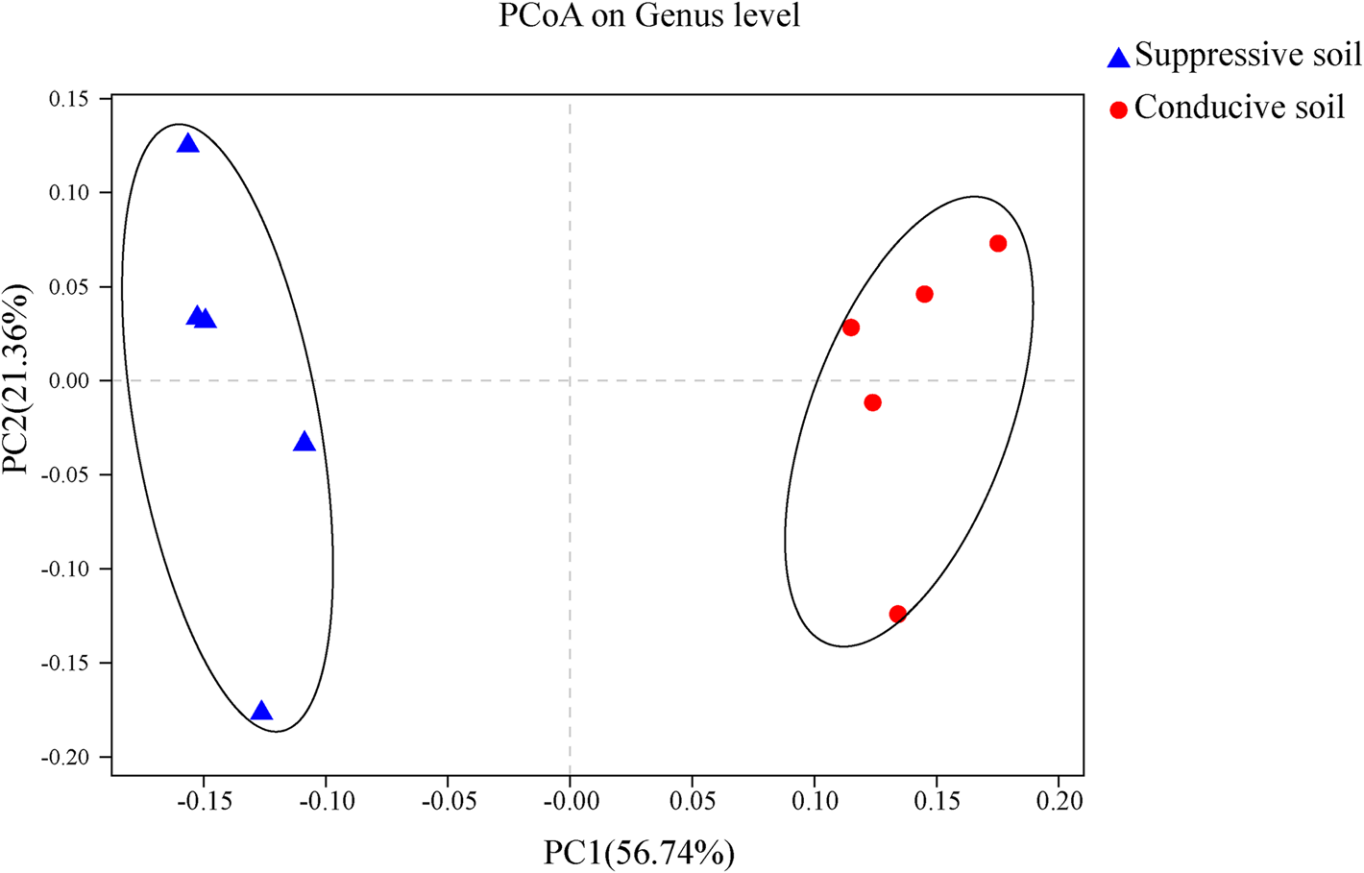
PCoA analysis at the genus level between suppressive soil (SS) and conducive soil (CS). The β-diversity was calculated based on the Bray-Curtis algorithm

**Fig. 4:**
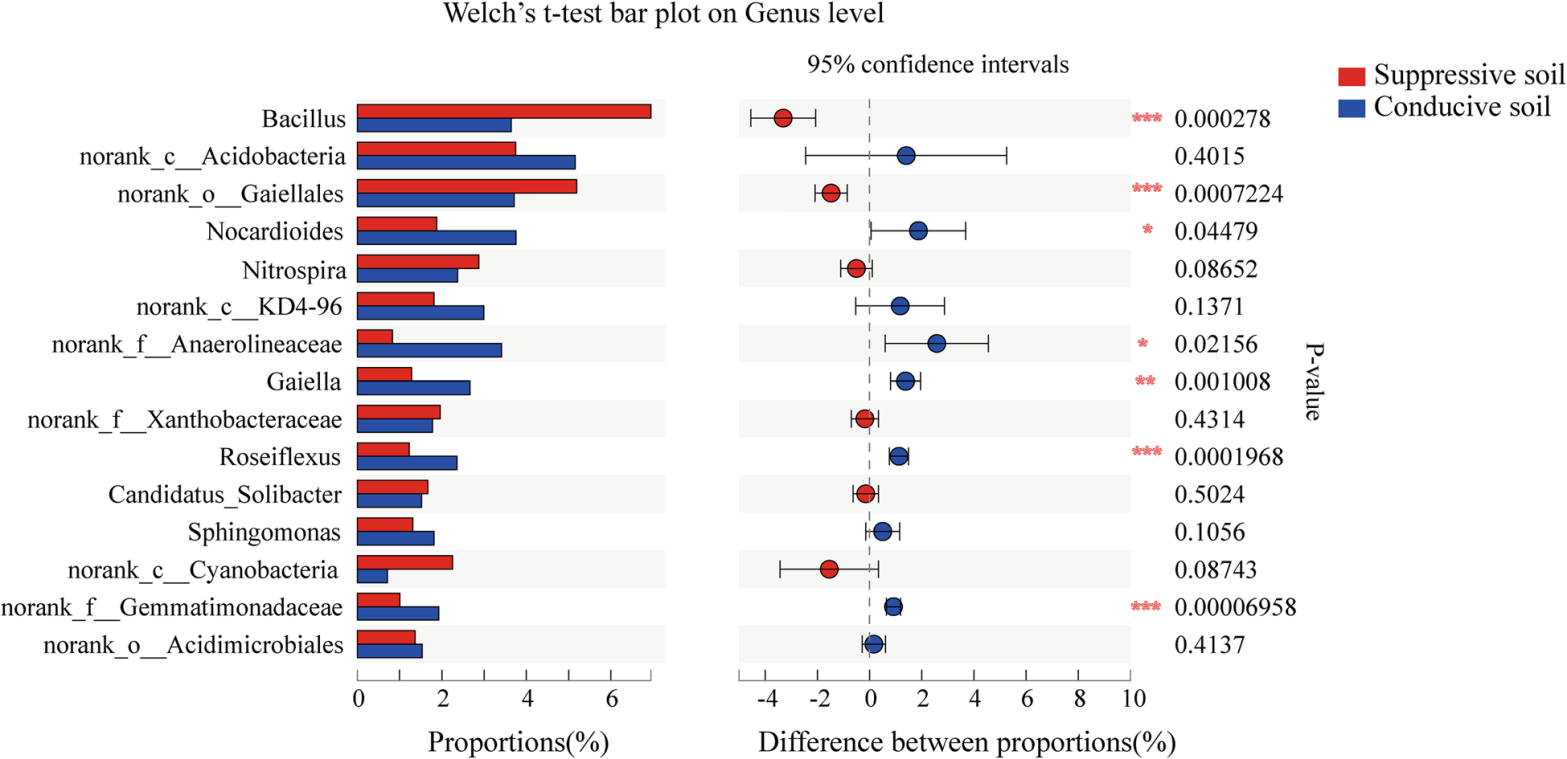
Welch’s *t*-test and FDR comparison analysis of microbial communities at the genus level between suppressive soil (SS) and conducive soil (CS). The Scheffe’s value cutoff was 0.95, ***p ≤ 0.001, **0.001 < *p* ≤ 0.01, and *0.01 < *p* ≤ 0.05

The primary bacterial genera of the SS samples were *Bacillus* (relative abundance 7.18%), *norank_o*_*Gaiellales* (5.20%), *norank_c*_*Acidobacteria* (3.28%), *Nocardioides* (2.31%), *Nitrospira* (2.74%), *norank_c_KD4–96* (1.82%), *norank_f*_*Xanthobacteraceae* (1.99%). The genera *Bacillus* (3.95%), *norank_o*_*Gaiellales* (3.94%), *norank_c_Acidobacteria* (3.76%), *Nocardioides* (3.77%), *Gaiella* (2.96%) *norank_c_KD4–96* (2.47%), *Roseiflexus* (2.49%), and *norank_f*_*Anaerolineaceae* (2.41%) were the dominate groups of CS samples (Fig. 2). In addition, principal coordinates analysis (PCoA) revealed that the bacterial communities of SS and CS were distinct, and the PC1 axis showed 56.74% variation in the bacteria community between SS and CS (Fig. 3). The relative abundance of genera *Nocardioides*, *Gaiella* and *norank_f_Anaerolineaceae* between SS and CS reached significant (95% confidence interval, CI; 0.01 < *p* < 0.05). In addition, the relative abundance of genera *Bacillus*, *norank_o_Gaiellales*, *Roseiflexus*, and *norank_o*_*Gemmatimonadaceae* were significant (95% CI, *p* < 0.01; Fig. 4).

### Chemical properties and redundancy analysis (RDA) of SS and CS

**Fig. 5:**
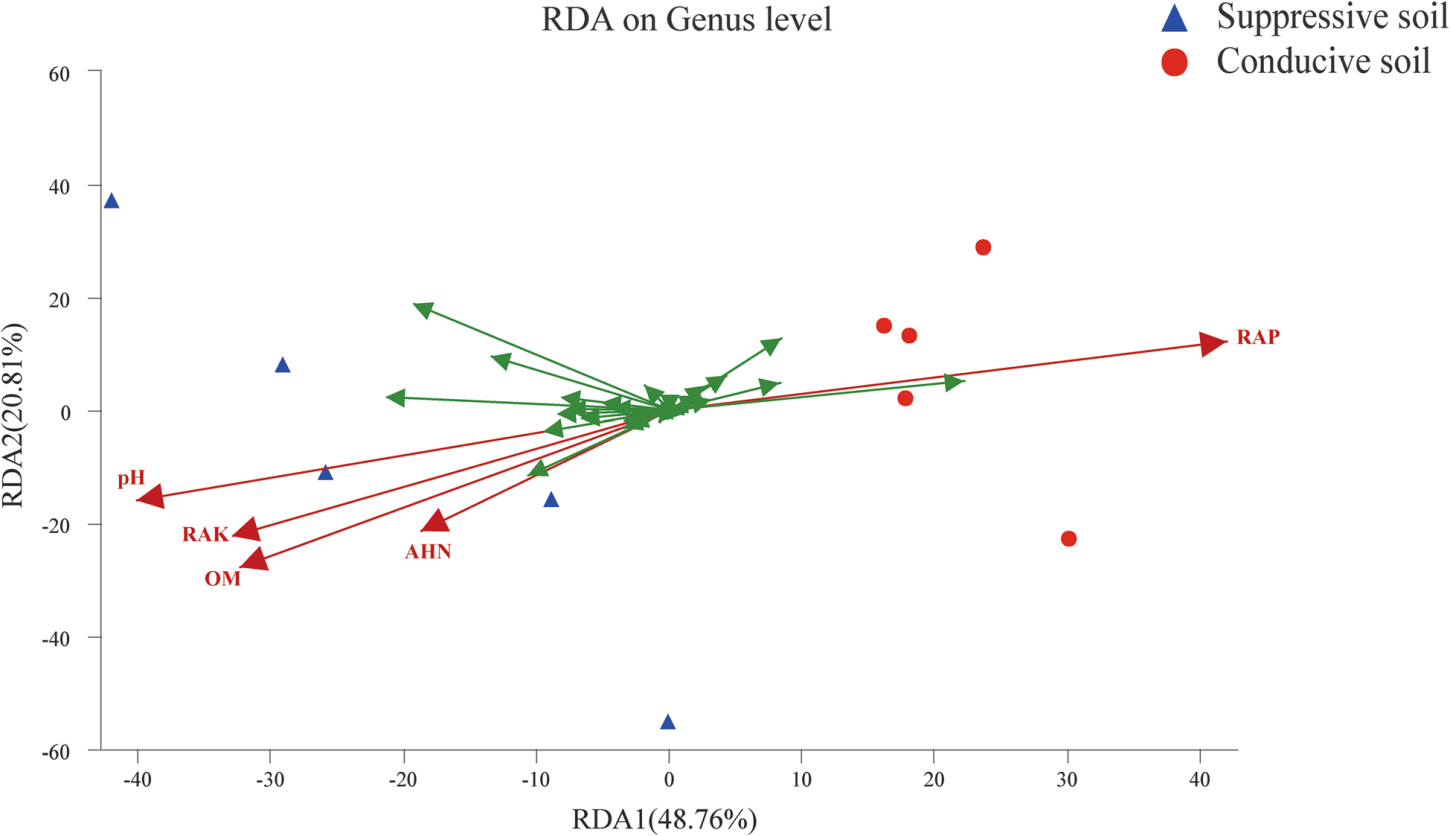
Correlations analysis between bacterial diversity (genus level) and soil chemical properties of suppressive soil (SS) and conducive soil (CS). The green arrows represent genus, red arrows represent environmental factors (EF): soil pH, organic matter (OM), alkali-hydrolyzable nitrogen (AHN), rapidly available phosphate (RAP), and rapidly available potassium (RAK). The length of the red arrows indicates the degree of impact by related EF, the angle between EF represents positive or negative correlation, the acute angle represents positive correlation, the obtuse angle represents negative correlation, and the right angle represents no correlation

No significant difference was observed for alkali-hydrolyzable nitrogen (AHN) content of the SS and CS samples. The pH, organic matter (OM) content, and rapidly available potassium (RAK) of SS were significantly lower than the CS sample (*p* < 0.05, Table 1), but the content plant rapidly available phosphate (RAP) in SS was significantly higher than CS. RDA analysis at genus level revealed that RAP played a key role in the difference in bacterial community distribution between SS and CS, and it was negatively correlated with the other four chemical contents (Fig. 5).

**Table 1:** The suppressive and conducive soil chemical properties

Properties	Healthy soils (***n*** = 5)	Diseased soils (***n*** = 5)
Location	N 18^。^29′51″, E 109^。^56′54″	N 18^。^32′47″, E 110^。^3′1″
pH	5.19 ± 0.16	6.64 ± 0.14 **
OM (%)	0.85 ± 0.13	1.15 ± 0.082 *
RAP (mg/kg)	152.02 ± 10.37	86.95 ± 5.58 **
RAK (mg/kg)	102.34 ± 8.46	247.69 ± 54.68 **
AHN (mg/kg)	35.77 ± 3.18	39.21 ± 2.82

### Network analysis of SS and CS

**Fig. 6:**
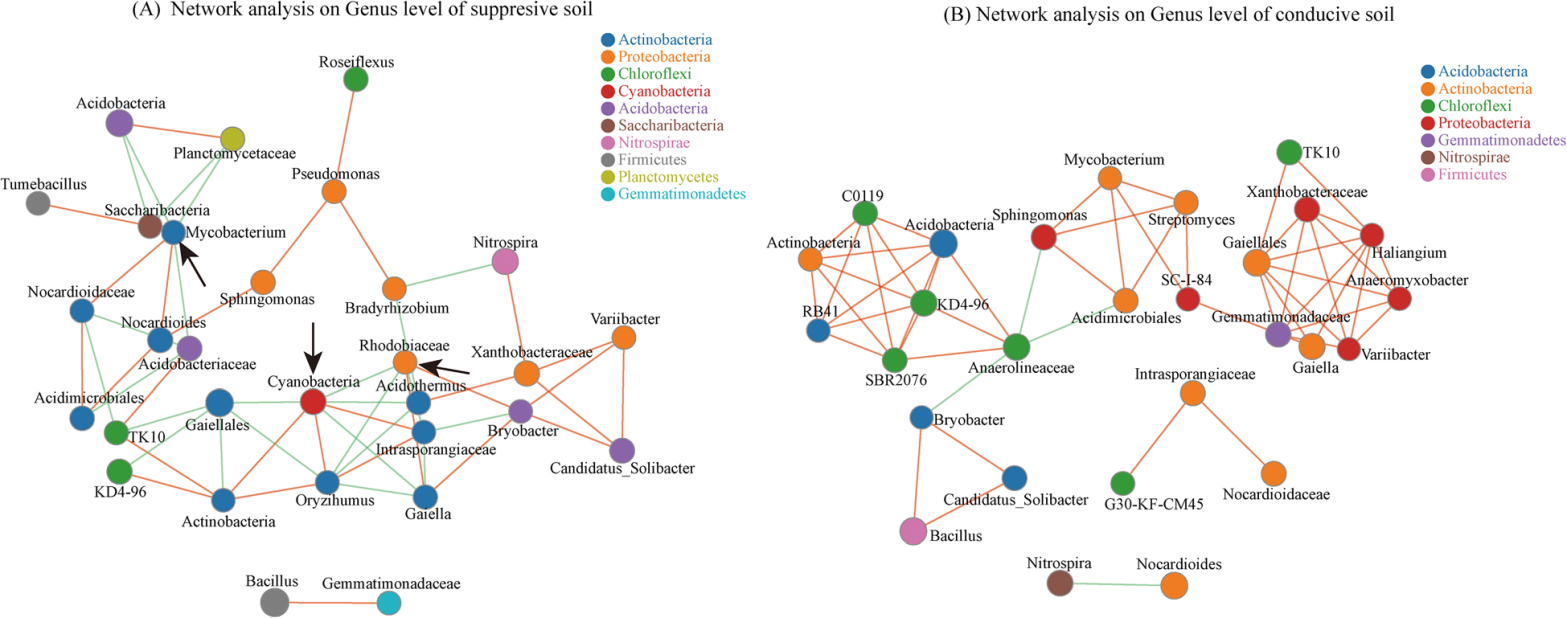
Interaction network analysis at the genus level of suppressive soil (SS) **a** and conducive soil (CS) **b**. Different node colors represent different bacteria genera. Blue lines represent negative interactions; red lines represent positive interactions among different nodes

Network analysis of the 30 most abundant genera revealed the interaction relationships of SS and CS, respectively. The results indicated that there were extensive interactions among the identified genera. In the SS (Fig. 6a), the 30 most abundant genera were from 10 phyla, including 10 genera from Actinobacteria, six genera from Proteobacteria, four genera from Acidobacteria, three genera from Chloroflexi, two genera from Firmicutes, and one genus each from Nitrospirae, Saccharibacteria, Planctomycetes, Cyanobacteria, and Gemmatimonadetes, respectively. Bacteria from *Mycobacterium*, *Rhodobiaceae* and *Cyanobacteria* showed interaction relationships with five, seven, and seven other genera, respectively. In the CS samples (Fig. 6b), these genera were only from seven phyla, such as the nine genera from Actinobacteria, six genera from Proteobacteria, six genera from Chloroflexi, four genera from Acidobacteria, one genus from Gemmatimonadetes, Nitrospirae, and Firmicutes, respectively.

### Analysis of the COGs with significant differences

**Fig. 7:**
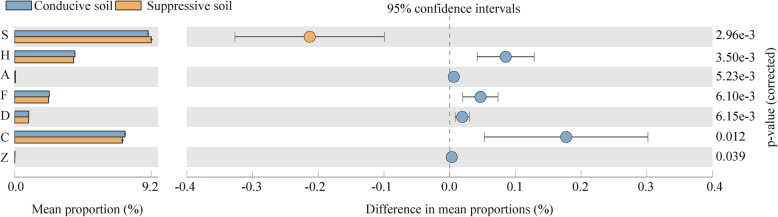
Significantly different COGs between suppressive soil (SS) and conducive soil (CS). S: function unknown; H: coenzyme transport and metabolism; A: RNA processing and modification; F: nucleotide transport and metabolism; D: cell cycle control, cell division, chromosome partitioning; C: energy production and conversion; Z: cytoskeleton

COG function prediction was performed and compared between SS and CS samples. The COGs with significant differences were further investigated. There were seven COGs that were significantly different, such as the group S (function unknown), H (coenzyme transport and metabolism), A (RNA processing and modification), F (nucleotide transport and metabolism), and D (cell cycle control, cell division, chromosome partitioning). The groups C (energy production and conversion) and Z (cytoskeleton) were very distinct (Fig. 7).

### Isolation and identification of antagonistic strains

Few tomato plants in the CS field grew well. We collected the rhizosphere soil of three healthy tomato plants and isolated the cultural bacteria. Using the inhibition zone method, 55 bacterial strains with distinct antagonistic activities against *R. solancearum* strain EP1 were identified by sequencing their 16S *rDNA* sequences (Table 2). The basic local alignment search tool (BLAST) results showed that these strains belong to the genera *Bacillus* (17 strains), *Pseudomonas* (10 strains), *Sphingobacterium* (10 strains), *Chryseobacterium* (9 strains), *Serratia* (four strains), *Cellulosimicrobium* (one strain), *Staphylococcus* (one strain), *Fictibacillus* (one strain), *Microbacterium* (one strain), and *Paenibacillus* (one strain)*.*

**Table 2:** The antagonistic strains against *R. solanacearum* strain EP1

Genus	Strain number	GenBank accession No.
*Bacillus*	17	MN410647–48, 51–59, 61–62,71,74, 98, 70
*Pseudomonas*	10	MN410664–65, 68–69, 75–76, 79, 89, 92, 94
*Sphingobacterium*	10	MN410666–67, 72–73, 81–82, 85–86, 93, 96
*Chryseobacterium*	9	MN410677, 80, 83–84 87–88, 90–91, 95
*Serratia*	4	MN410670, 78, 91, 99
*Staphylococcus*	1	MN410649
*Fictibacillus*	1	MN410650
*Microbacterium*	1	MN410660
*Cellulosimicrobium*	1	MN410663
*Paenibacillus*	1	MN410701

## Discussion

Previous studies have compared the microbial species abundance and diversity of bacterial wilt disease outbreak in soil samples [16–18]. Here, the microbial relative abundances and diversities of SS and CS were determined in addition to the associated soil chemical properties, microbial networks and COG groups with significant differences. Notably, we also isolated and identified 55 bacteria strains with excellent antagonistic activities to *R. solanacearum* strain EP1.

Results of Sobs index of the OTU level, main bacterial community distribution and PCoA indicated that significant differences in bacterial community composition exist between SS and CS (Figs. 1-3). Based on the result of Welch’s t-test (95% CI) at the genus level, the relative abundances of *Bacillus*, *norank_c_Gaiellales*, *Roseiflexus*, and *norank_f*_*Gemmatimonadaceae* were statistically significant (Fig. 4). Thus, the relative abundances of these four genera changed distinctly as bacterial wilt progressed. Numerous studies have proved that the *Bacillus* species are beneficial microorganisms, producing a vast array of biologically active molecules that inhibit pathogens [19]. The cyclic lipopeptides antimicrobial compounds produced by *Bacillus* species, such as surfactin, iturin, and fengycin, have been well studied and applied for their antagonistic activities in reducing diseases caused by *R. solanacearum* [20], *Rhizoctonia solani* [21], *Pythium aphanidermatum* [22], and *Podosphaera fusca* [23]. In this study, the relative abundances of *Bacillus* in SS samples were higher than that in CS samples. Moreover, the result of antagonistic strain isolation and identification from the healthy tomato plant roots was also supported these data, for 17 *Bacillus* strains were found among the 55 antagonistic strains. These results further demonstrate that the *Bacillus* species is one key bacterial group for protecting plant health. In addition, the strains of *Sphingobacterium*, *Chryseobacterium*, *Serratia*, *Cellulosimicrobium*, *Staphylococcus*, *Fictibacillus*, *Microbacterium*, and *Paenibacillus* were also found with antagonistic activity against *R. solanacearum* in our study, however, based on our best understanding, which were not so extensive reported. Thus, our study provides new resources for future biological control of bacterial wilt.

Soil is considered a highly complex and dynamic ecosystem [24], and the results of a recent study demonstrate that the formation of disease suppressive soils after a disease outbreak is likely due to the subsequent assembly of beneficial microbiota in the plant rhizosphere [25]. Network analysis can provide information regarding the symbiotic relationships of species in environmental samples, as well as species interactions and mechanisms of formation of phenotypic differences between samples. Our network analysis results of SS and CS samples showed that greater genus community diversity and more complex interactions existed in the SS samples. The groups of phyla Cyanobacteria, Saccharibacteria and Planctomycetes were not found in the interaction network of the CS sample, and several lonely interaction units were formed in CS network (Fig. 6). Thus, the nodes in the SS network analysis were connected with more interactions than in the CS network, forming a stable network. In particular, the species of *Mycobacterium*, *Cyanobacteria* and *Rhodobiaceae* exhibited extensive interactions to other genera, which should be the key components that sustain the stable network, and *Cyanobacteria* has been confirmed to show extensive interactions with soil microbiota and play key roles in shaping the course of evolution and ecological change [26].

It is worth noting that among the main genera groups of SS and CS, there were 10 and 9 genera in the phylum Actinobacteria in SS and CS, respectively. Obviously, the phylum of Actinobacteria with important niches in SS and CS samples. Strains of Actinobacteria are effective biocontrol microorganisms against many plant pathogens, including *R. solanacearum* [27, 28]; however, once these strains colonize on the plant surface, which can also inhibit other beneficial microbes for their broad-spectrum antimicrobial compounds [29]. Thus, they can also negatively impact the bacterial community. By comparing the network analysis between SS and CS, we found that several genera are negatively correlated with the groups of Actinobacteria in SS network, such as the genera from Chloroflexi, Cyanobacteria, Planctomycetes, and Acidobacteria, as well as some genera of Proteobacteria (*Bradyrhizobium*, *Rhodobiaceae*). They can balance or limit the antagonistic effects of Actinobacteria to other microorganisms, and subsequently help construct a more stable bacterial community with greater diversity. Previous results have shown that the soil with greater bacterial community diversity facilitates microorganism nutrient cycling, promotes plant growth, adapts to environmental changes and suppresses plant pathogens [30, 31]. However, in the CS network, a significant positive correlation existed between Actinobacteria and other genera; thus, it is unfavorable to sustain the bacterial community diversity, especially once the plant-beneficial groups were inhibited, which may lead to CS fail to prevent the infection of RSSC. Thus, the main reasons for outbreak of bacterial wilt include a decline in the number of plant-beneficial groups, decreasing bacterial diversity and the accumulation of plant pathogens. Conversely, a stable and complex network existed in SS, helping to prevent RSSC infection and sustain the tomato plant health.

It has been demonstrated that pH has a significant impact on bacterial abundance [32]. Some related reports predicted that soil acidification could negatively affect the bacterial community stability, leading to bacterial wilt [33]. However, we found that the pH was lower in SS samples than in CS; therefore, other soil chemical properties may contribute to the stability of the soil bacterial community or the susceptibility to RSSC. Interestingly, we found that RAP played a vital role in the difference in the bacterial community distribution between SS and CS. Additionally, a negative correlation with the other four chemical factors was found. Thus, the RAP may function differently from other chemical factors (Fig. 5).

## Conclusions

The results of this study show that the microbial diversities were quite different between *R. solanacearum* resistant and *R. solanacearum* susceptible soils. Moreover, RAP played a key role in the difference in bacterial community distribution and was negatively correlated with pH, OM content, AHN, and RAK. Interaction network analysis further demonstrated that greater microbial diversity led to more extensive interactions in SS and, subsequently, a stable network. Importantly, we isolated and identified 55 bacteria strains with excellent antagonistic ability to *R. solanacearum* from the rhizosphere soil of healthy tomato plants collected from CS. This study provides a good foundation for future biological control of bacterial wilt.

## Methods

### Collection and DNA extraction of soil samples

Soil samples were collected from Benhao town, Lingshui country, Hainan province, China, and the collection of all of the soil samples was permissible by Lingshui country government. The SS samples were located at N: 18^°^29′51″, E: 109^°^56′54″. The CS samples were located at N: 18^°^32′47″, E: 110^°^3′1″ (Table 1). At these particular sites, cherry tomato has been continuously planted more than 5 years. First, grass at the surface was cleared in a 1 m × 1 m square. The soils from 10 cm depth of three different locations were combined as one soil sample. Five replicate mixed soil samples were collected from healthy and diseased fields, respectively. All of the samples were stored in sterile plastic bags and transported to the laboratory in an icebox immediately. The samples were stored at − 20 °C until *16S rDNA* sequencing and analysis.

Aliquots (0.25 g) of the soil samples were processed using a MOBIO PowerSoil® kit. The extracted DNA samples were analyzed using a NanoDrop 2000 UV-Vis spectrophotometer (Thermo Scientific, Wilmington, DE, USA). The DNA quality was confirmed by 1% agarose gel electrophoresis. The extracted DNA samples were selected and used to conduct microbial community analysis by PCR using the following *16S rDNA* primers: forward (5′-GTGCCAGCMGCCGCGG-3′) and reverse (5′-CCGTCAATTCMTTTRAGTTT-3′) [34]. The PCR reactions were conducted as follows: 95 °C for 3 min; followed by 27 cycles of 30 s at 95 °C; 30 s at 55 °C; 72 °C for 45 s; and a final extension at 72 °C for 10 min. The PCR reactions were performed in triplicate, using 20 μL mixtures containing 4 μL 5× FastPfu buffer, 2 μL 2.5 mM dNTPs, 1 μL primer mix (5 μL), 0.4 μL FastPfu polymerase, and 5 ng extracted DNA as the template. The PCR products were extracted from a 2% agarose gel and further purified using the AxyPrep DNA Gel Extraction Kit (Axygen Biosciences, Union City, CA, USA). The products were quantified using QuantiFluor-ST (Promega, Madison, USA). Purified amplicons were then pooled in equimolar concentrations and paired-end sequenced (2 × 300) using the Illumina MiSeq platform (Illumina, San Diego, CA, USA) according to the standard protocols of Shanghai Majorbio Bio-pharm Technology Co., Ltd. Raw sequences were filtrated using FASTX Toolkit 0.0.12 software to remove low quality reads with Q value < 20 and less than 35 bp [33].

### Diversity analysis of microbial communities

*16S rDNA* data were analyzed using the Majorbio I-Sanger cloud platform (http://www.i-sanger.com). The similarity and differences between samples were compared using the shared and unique OTU of a Venn diagram, and Student’s t-test was used to assess the level of significant difference. The bar and pie analyses were conducted at the genus level. The PCoA of β-diversity was calculated based on the Bray-Curtis algorithm. To compare significance testing of microbial community variance of SS and CS samples at the genus level, Welch’s t-test (two-tailed test) and false discovery rate were used with a Scheffe cutoff value of 0.95. The Welch’s inverted confidence interval method was used to calculate the CI value. Network analysis was conducted at the genus level to assess the correlation characterization of the SS and CS samples, and the 30 most abundant OTUs were selected. For these analyses, the Spearman correlation coefficient model was used with a cutoff of 0.5. To conduct the *16S rDNA* function prediction, the richness of OTU was standardized by PICRUSt, and, subsequently, the COG family information corresponding to OTU and COG richness was obtained. The COGs with significant differences were analyzed by Stamp software (the statistical test model was an ANOVA and post hoc test model was a Tukey-Kramer with a cutoff 0.95).

### Correlation between soil chemical characteristics and bacterial diversity

Soil pH, OM content, AHN, RAP, and RAK were determined as previously reported [33]. RDA was conducted to calculate the bacterial diversity distribution correlation with the above soil chemical properties at the genus level.

### Isolation and identification of antagonistic bacteria

Rhizosphere soils from healthy tomato plant roots planted in CS were collected. The root tissues were shredded and ground fully with the collected rhizosphere soils. Fifty milliliters of sterile water were added to the above collecting pipe. After a series of gradient dilutions, the suspensions were spread on CN agar medium (0.1% casamino acid, 0.1% nutrient broth, 1% agar). The dilution that resulted in 40–60 colonies per plate was selected. The colonies with different phenotypes were separated and purified. The inhibition zone method was used to assess the inhibition ability of each strain. The method is described briefly as follows: each isolated strain was cultured in the CN liquid medium (about 72 h, 28 °C, 200 rpm). The *R. solanacearum* strain EP1 [35] was selected as an indicator strain. It was cultured in CPG medium (casamino acid 1‰, peptone 1%, glucose 1%) until the OD_600_ was 1.0. One milliliter of EP1 suspension was added to 100 mL CPG agar medium (melting but not burning) and mixed well. Four microliters of the suspension of isolated rhizosphere strain were dispensed on the CPG plate with added EP1 suspension. The cells were cultured in an incubator at 28 °C. The diameters of the inhibition zone were recorded and analyzed after 3 days. All of the strains with inhibition ability were identified with *16S rDNA* sequencing (27F/1492R), and the corresponding genus was determined based on the BLAST results of *16S rRNA* sequence database (Bacteria and Archaea).

### Assessment of microbial function of soil samples

The SS and CS were collected and used to assess microbial function. In this study, the CS soil underwent heat treatment (autoclaving, 121 °C for 1 h, followed by dry heat sterilization, 180 °C for 4 h) as the control. In addition, sterile nutriment soil was inoculated with the *R. solanacearum* suspension. Five milliliters of *R. solanacearum* strain EP1 suspension (OD_600_ ≈ 1.0) and 100 mL sterile water were applied to the root soil. All of the plants were placed into a climate-controlled room with a 14 h/10 h light/dark cycle at 28 °C. Plants were monitored for disease progression over time after inoculation, and bacterial wilt rates were recorded. The inoculation experiments were repeated three times. Tomato plants (cultivar: Qianxi cherry tomato) were used in the above treatments.

## Supplementary information

**Additional file 1 S1.** Disease incidence of tomato plants cultivated with suppressive soil (A), conducive soil with heat treatment (B), conducive soil (C), and sterile nutrition soil inoculated with *R. solanacearum* suspension (D).

## Data Availability

The raw reads of 16S MiSeq data were deposited into the NCBI Sequence Read Archive database (accession No.: PRJNA564488). All the 16S rDNA sequences of 55 antagonistic strains have been upload to NCBI database (GenBank accession no. MN410647 - MN410701).

## Abbreviations

AHN: Alkali-hydrolyzable nitrogen
BLAST: Basic local alignment search tool
CI: Confidence interval
CS: Conducive soil
CN: Casamino acid-nutrient broth agar
COGs: Clusters of orthologous groups
CPG: Casamino acid - peptone - glucose
OM: Organic matter
OTUs: Operational taxonomic units
RAK: Rapid available potassium
RAP: Rapidly available phosphate
RDA: Redundancy analysis
RSSC: *Ralstonia solanacearum* species complex
SS: Suppressive soil
