# Supplementary figures and images for: Comparison of bacterial communities in soil samples with and without tomato bacterial wilt caused by Ralstonia solanacearum species complex
Source: BMC Microbiol. 2020 Apr 14;20:89. doi: 10.1186/s12866-020-01774-y (PMC7155298; doi:10.1186/s12866-020-01774-y)

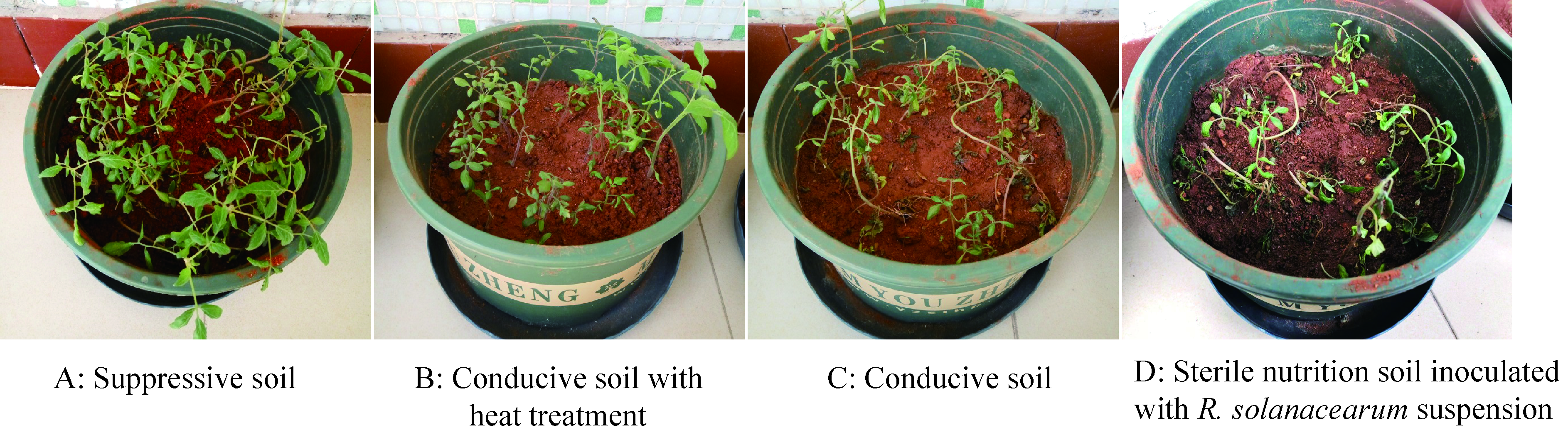

Supplement: Supplementary file 1 — Additional file 1 S1. Disease incidence of tomato plants cultivated with suppressive soil (A), conducive soil with heat treatment (B), conducive soil (C), and sterile nutrition soil inoculated with R. solanacearum suspension (D). [file 12866_2020_1774_MOESM1_ESM.tif]
